# A 4′‐Thioribouridine Modification Mimics 2′‐*O*‐Methoxyethyl in ASO‐Like 20‐Mers Targeting *PTEN* Knockdown

**DOI:** 10.1002/cbic.70533

**Published:** 2026-09-27

**Authors:** Joanna E. Parkes, Caecilie M. M. Benckendorff, Anna L. Malinowska, Jacob Roberts, Harley L. Huynh, Sritama Bose, Loïc M. Cochard, Aisling Ní Cheallaigh, Sarah L. Lovelock, Peter L. Oliver, Gavin J. Miller

**Affiliations:** ^1^ MRC Nucleic Acid Therapy Accelerator (NATA) Research Complex at Harwell (RCaH) Oxford UK; ^2^ Manchester Institute of Biotechnology & Department of Chemistry University of Manchester Manchester UK

**Keywords:** aptamer, gene knockdown, nuclease, oligonucleotide, phosphoramidite, PTEN, small interfering RNA

## Abstract

Gapmer antisense oligonucleotides (ASOs) represent a clinically validated therapeutic platform, with a 2′‐*O*‐methoxyethyl modification commonplace within the flanking regions. An alternative sugar modification, incorporation of 4′‐thioribose, has demonstrated enhanced nuclease resistance in siRNAs and aptamers, but incorporation of this motif into gapmer architectures remains underexplored. Herein, we report the synthesis of a 4′‐thiouridine phosphoramidite building block and its incorporation into gapmer ASOs targeting *PTEN*. Evaluation of sequences containing 4′‐thiouridine in the flanking regions (compared with 2′‐*O*‐methoxyethyl) and varying phosphorothioate linkage composition demonstrates that the modified gapmers maintain equivalent knockdown efficacy under transfection and gymnotic delivery. These findings indicate 4′‐thioribose as an alternative sugar modification for wider exploration within gapmer ASOs.

## Graphical abstract

A 4′‐thiouridine phosphoramidite was prepared and site‐selectively incorporated into *PTEN*‐targeting gapmer ASOs. Flank‐modified gapmers with defined phosphorothioate linkage patterns maintained potent target knockdown under transfection and gymnotic delivery, matching 2′‐*O*‐methoxyethyl comparators and highlighting 4′‐thioribose as a valuable motif for ASO design.

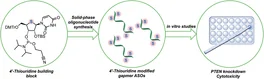

## 1 Introduction

Structurally modified nucleosides provide cornerstone building blocks for derived oligonucleotide (or nucleic acid) therapeutics that commonly target mRNA to modify the production of disease‐related proteins [1, 2]. Oligonucleotide therapeutics are an emergent drug modality and consist of modified or unmodified short nucleic acid sequences; these include antisense oligonucleotides (ASOs), small interfering RNAs (siRNAs), microRNA (miRNAs), aptamers and DNAzymes [3]. Currently, >24 oligonucleotide therapeutics have been granted new drug approval by the U.S. Food and Drug Administration (FDA), and many more are in the advanced stages of clinical trials [4, 5]. Gapmer ASOs employ a strategic architecture in which modified flanking regions provide nuclease resistance and binding affinity, while a central DNA segment recruits RNase H to catalyse degradation of the target mRNA [6].

Crucially, chemical modifications have been developed to confer favourable pharmacological properties to nucleic acid therapeutics, including improved resistance to nuclease degradation, increased binding affinity and reduced immune response [6]. Commonly, modifications are made to the 2′‐position of the ribose sugar and include 2′‐*O*‐methyl (2′‐OMe), 2′‐fluoro and 2′‐*O*‐methoxyethyl (2′‐MOE) [4, 7]. Constrained modifications such as locked nucleic acids (LNA), in which a methylene bridge connects the 2′‐oxygen to the 4′‐carbon, have also been shown to impart highly favourable properties by dramatically increasing binding affinity through conformational preorganisation [8]. Notably, the 2′‐MOE modification has emerged as the preferable modification within oligonucleotide flanking regions, demonstrated through the approved drugs mipomersen, inotersen and tofersen, where 2′‐MOE ‘wings’ provide stability to exonuclease degradation while maintaining favourable pharmacokinetic and toxicological properties [4, 9]. The clinical success of 2′‐MOE‐containing gapmers has demonstrated that the appropriate chemical modification of flanking regions is essential for achieving nuclease resistance and the prolonged half‐life required for therapeutic efficacy. Importantly, such modifications must be optimised to minimise both sequence dependent toxicities (arising from unintended target engagement) and sequence‐independent toxicities, such as immune activation, complement activation or organ‐specific adverse effects [4].

**FIGURE 1:**
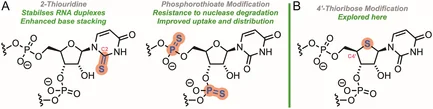
(A) Heteroatom modifications to the nucleobase and phosphate backbone within oligonucleotide sequences. (B) Sulphur substitution of D‐ribose oxygen explored in this study.

Beyond ribose 2′‐position modifications, heteroatom substitution represents another important strategy for nucleic acid therapeutic development. Replacement of canonical oxygen atoms using sulphur within nucleobases and phosphate ester linkages has given rise to successful thiouridine and phosphorothioate (PS) modifications, with such changes improving hydrolytic stability and enriching target specificity (Figure 1A) [10, 11]. Sulphur inclusion within the ribose sugar (Figure 1B), while reported by a number of academic groups and demonstrating important biological activity [4, 12, 13, 14, 15, 16, 17, 18, 19], remains comparatively underexplored and lacks wider uptake, likely due to challenges in efficiently synthesising the required building blocks.

Imbach and colleagues reported in 1993 that 4′‐thio‐oligouridylates exhibited remarkable nuclease resistance while maintaining biological activity [16], with subsequent studies confirming resistance to multiple nucleases [17]. Incorporation of 4′‐thionucleosides into RNA strands containing 2′‐*O*‐alkyl modifications (2′‐OMe and 2′‐MOE) was shown to significantly improve siRNA activity and nuclease stability in mammalian cells [20]. Structural characterisation by Egli and coworkers demonstrated that 4′‐thiocytosine incorporation enhanced thermodynamic stability with minimal structural perturbation to the oligonucleotide [14]. Compatibility of this heteroatom modification with enzymatic synthesis methods was also established through successful incorporation of 4′‐thioNTPs into RNA aptamers via SELEX [15, 18], with studies confirming thermodynamic stability of 4′‐thioRNA duplexes [18]. Furthermore, 2′‐modifications (2′‐F and 2′‐OMe) of 4′‐thio‐containing RNA have demonstrated increased thermodynamic and nuclease stability [13], and recently a 4′‐thio LNA modification was shown to enhance hybridisation and exonuclease resistance [12].

Despite these promising characteristics and demonstrated utility of a 4′‐thio modification in siRNA and aptamer applications, their incorporation into gapmer ASO architectures remains underexplored. Furthermore, the relationship between a 4′‐thio modification pattern in gapmer wings, sequence‐specific mRNA targeting efficiency and cellular toxicity profiles has not yet been evaluated. In this study, we investigate the incorporation of a 4′‐thiouridine modification into gapmer ASOs targeting *PTEN*. By replacing canonical uridine with 4′‐thiouridine in the flanking regions and varying the phosphorothioate backbone content throughout derived gapmer ASO 20‐mers, we assess the impact on target knockdown efficiency and cellular toxicity and compare this heteroatom modification to the established 2′‐MOE system.

## 2 Results and Discussion

### 2.1 Synthesis of 4′‐Thiouridine‐Containing Gapmers

**FIGURE 2:**
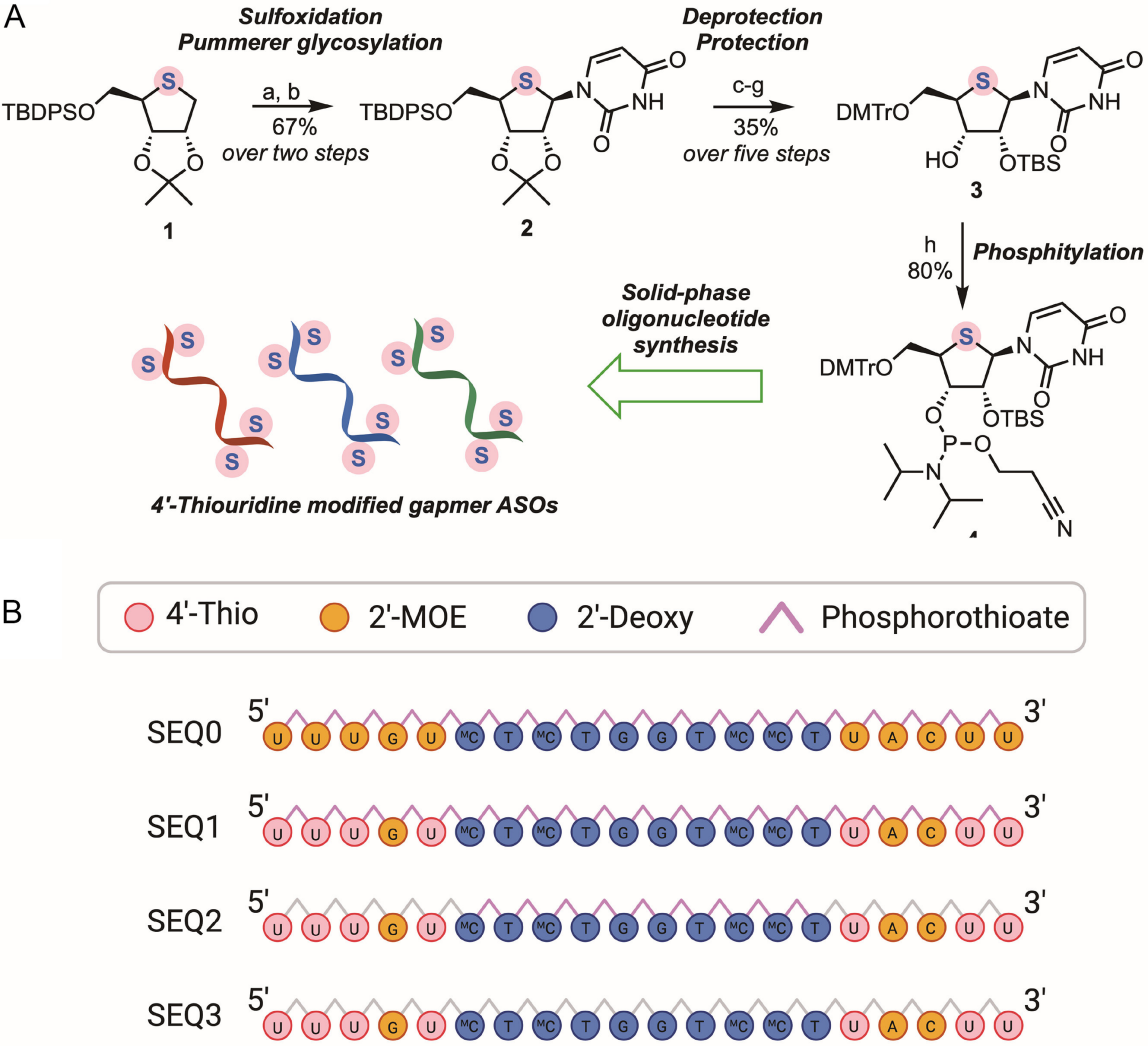
Synthesis of 3′‐*O*‐phosphoramidite building block **4** and the gapmer ASO sequences synthesised therefrom. (A) Reagents and conditions: (a) *m*‐CPBA, Et_2_O, 0 °C, 1 h; (b) (i) Uracil, HMDS, TMSCl, MeCN reflux, 1 h, (ii) **2**, TMSOTf, DIPEA, MeCN, 0 °C, 18 h; (c) TBAF, THF, rt, 45 min; (d) DOWEX 50W X8 H^+^, MeOH, H_2_O, reflux, 4 h; (e) (i) ^*t*^Bu_2_Si(OTf)_2_, 2,6‐lutidine, DMF, −18 to 0 °C, 8 h, (ii) TBSOTf, 0 °C to rt, 18 h; (f) HF·pyridine, pyridine, −10 °C, 3 h; (g) DMTrCl, pyridine, 18 h; (h) 2‐cyanoethyl diisopropylchlorophosphoramidite, DIPEA, THF, 0 °C, 1.5 h. (B) ASO gapmer sequences synthesised. Seq0 = positive control, 2′‐MOE gapmer sequence containing PS throughout (pink); Seq1 = 2’‐MOE uridine replaced with 4′‐thiouridine and containing PS throughout; Seq2 = 2′‐MOE uridine replaced with 4′‐thiouridine and phosphodiester bonds (grey) in the flanking regions; Seq3 = 2′‐MOE uridine replaced with 4′‐thiouridine and phosphodiester bonds throughout. G = guanosine, A = adenosine, C = cytidine, ^M^C = 5‐methylcytidine, T = thymidine, U = uridine/4′‐thiouridine.

Building upon our previous reports developing synthetic access to 4′‐thionucleosides [21, 22, 23, 24, 25], access to 4′‐thiouridine phosphoramidite building block **4** was achieved (Figure 2). Starting from 2.0 g of thioribitol derivative **1**, sulphoxidation followed by a Pummerer‐type glycosylation with uracil afforded protected 4′‐thiouridine **2** in 67% isolated yield over two steps, an improvement over the previously reported 50% yield [21]. This was realised by replacing the reaction solvent dichloromethane with diethyl ether during the oxidation step and then omitting it entirely from the glycosylation. Next, a sequence of appropriate protecting group manipulations delivered 4′‐thionucleoside **3**, with a further solvent modification, substitution of tetrahydrofuran for dichloromethane during the final 3′‐*O*‐phosphitylation reaction, increasing the isolated yield to 80%, and affording 400 mg of the desired 3′‐*O*‐phosphoramidite **4**. With building block **4** to hand, its incorporation into three 20‐mer sequences was performed using solid‐phase oligonucleotide synthesis (Seq1−3 Figure 2, see Supporting Information for details of SPOS).

### 2.2 A 4′‐Thiouridine‐PS Gapmer ASO Targeting *PTEN* Demonstrates Equivalent Cytotoxicity Compared With a Standard 2′‐MOE‐PS Gapmer ASO

**FIGURE 3:**
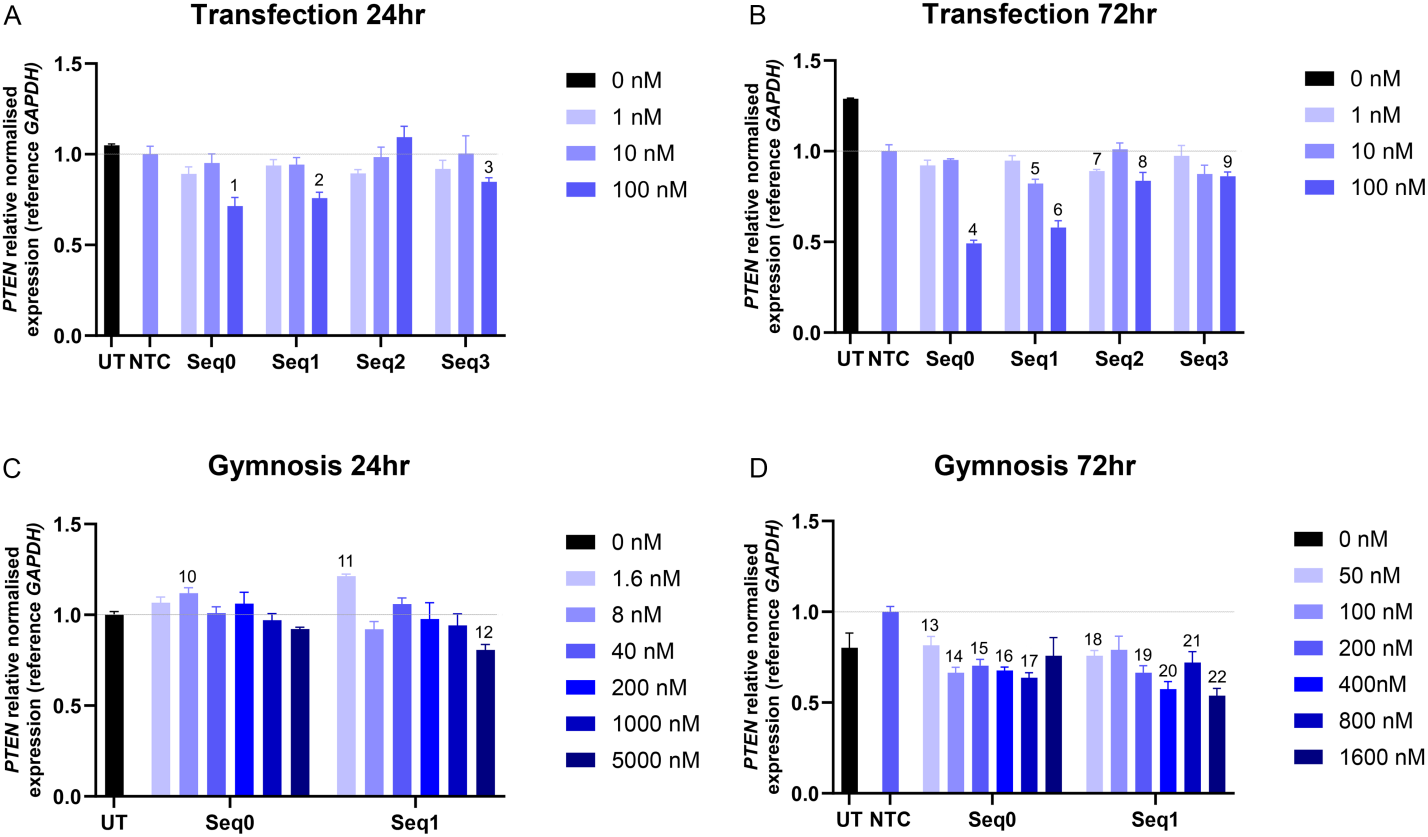
Relative normalised expression of *PTEN* following treatment with Seq0–Seq3 in HeLa cells. HeLa cells were transfected with the ASOs indicated for 24 (A) or 72 h (B) or treated by gymnosis for 24‐ (C) or 72 h (D). *PTEN* expression as measured by qRT‐PCR is shown relative to untreated cells (UT) or a non‐targeting control MOE gapmer (NTC) with all samples normalised to the reference gene *GAPDH*. Data are shown +SEM from N = 3 biological replicates. Numbers indicate significant difference versus the UT (24 h) or NTC (72 h) by 1‐way ANOVA with Dunnett’s multiple comparison test. *p* values: 1, 0.022; 2, 0.030; 3, 0.044; 4, 0.00087; 5, 0.0092; 6, 0.0051; 7, 0.048; 8, 0.042; 9, 0.033; 10, 0.026; 11, 0.0093; 12, 0.0078; 13, 0.029; 14, 0.0075; 15, 0.0061; 16, 0.0049; 17, 0.0017; 18, 0.023; 19, 0.00086; 20, 0.0069; 21, 0.043; 22, 0.0052.

To assess the impact of including 4′‐thionucleosides in a gapmer ASO, we selected an established 2′‐MOE sequence targeting *PTEN* that contained a significant proportion of uracil bases. In addition, the potency of this sequence had already been demonstrated in vitro ensuring that floor effects caused by saturating ASO activity were not expected [20]. To assess on‐target efficacy initially, transfection experiments were performed in parallel using four distinct sequences: the positive control 5‐10‐5 MOE‐PS gapmer with all phosphorothioate bonds (Seq0), with 4′‐thiouridine replacing 2′‐MOE uridine in the wings (Seq1), with 4′‐thiouridine replacing 2′‐MOE uridine and phosphodiester bonds in the wings (Seq2) and finally with 4′‐thiouridine replacing 2′‐MOE uridine in the wings and phosphodiester bonds throughout (Seq3, Figure 2B). After ASO transfection for 24 h at the highest dose (100 nM), Seq0 and Seq1 demonstrated significant on‐target knockdown of *PTEN* by approximately 25% when compared with the non‐targeting control (NTC) ASO gapmer (Figure 3A). Seventy‐two hours after ASO transfection, the activity of both Seq0 and Seq1 was more pronounced, with Seq1 showing a significant 18% reduction in *PTEN* expression at 10 nM, and both ASOs reaching approximately 50% knockdown at 100 nM (Figure 3B). For the PO‐containing ASOs, Seq2 and Seq3, some on‐target activity was detected at the highest doses, although only less than 12% (Figure 3A,B). Next, having demonstrated activity of the thionucleoside‐containing ASO Seq1, we assessed gymnotic activity of this compound against the standard Seq0 gapmer. After 24 h of exposure, only the highest dose began to show significant on‐target activity, with Seq1 reaching 19% knockdown versus 8% for Seq0. After 72 h, the level of *PTEN* was reduced significantly at almost all of the doses, with 1600 nM generating the greatest knockdown level at 46% with Seq1, while Seq0 reached a maximum of 36% at 800 nM (Figure 3C,D). The larger variability between replicates at 1600 nM for Seq0 (Figure 3D) may indicate some cytotoxicity at the higher doses for this ASO sequence.

**FIGURE 4:**
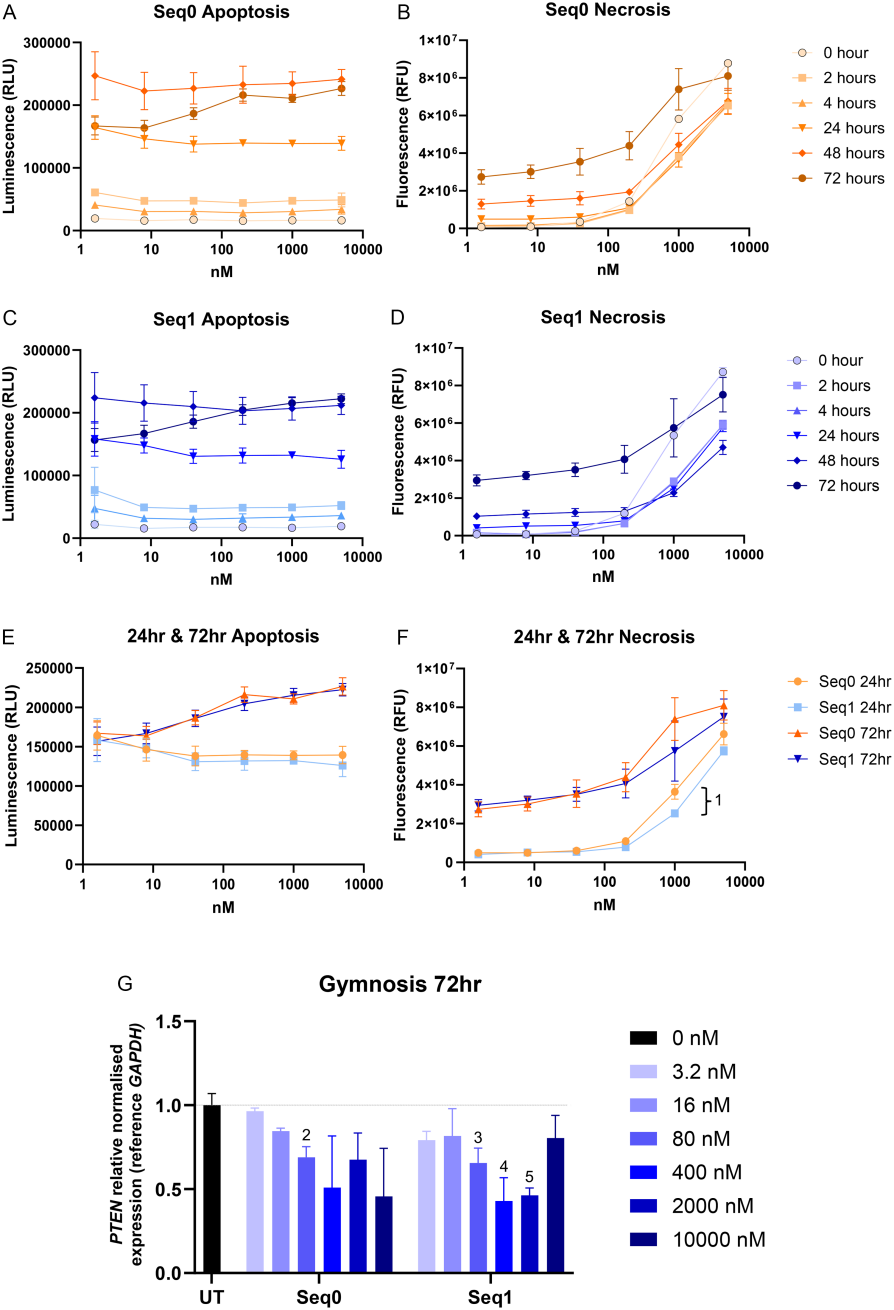
Cytotoxicity analyses of 2′‐MOE gapmers with and without uracil thionucleosides. (A) HeLa cells were treated with 2′‐MOE gapmers by gymnosis and luminescence or fluorescence signals were measured at 0, 2, 4, 24, 48 and 72 h to determine levels of apoptosis and necrosis, respectively. Normalised luminescence (apoptosis) for cells treated with Seq0 (A) or Seq1 (C) for all timepoints and a comparison of Seq0 and Seq1 at 24 and 72 h (E). Normalised fluorescence (necrosis) for cells treated with Seq0 (B) or Seq1 (D) for all timepoints and a comparison of Seq0 and Seq1 at 24 and 72 h (F). Data are shown +/− SEM from *N* = 4 biological replicates. Numbers indicate the significant difference between Seq0 and Seq1 at the 24‐ and 72‐h timepoints by 2‐way ANOVA. *p*‐value: 1: 0.048. (G) HeLa cells were treated by gymnosis for 72 h using Seq0 and Seq1 ASOs. *PTEN* expression as measured by qRT‐PCR is shown relative to untreated cells (UT) with all samples normalised to the reference gene *GAPDH*. Data are shown +SEM from N = 3 biological replicates. The number indicates the significant difference versus UT cells by 1‐way ANOVA with Dunnett’s multiple comparison test. *p*‐values: 2, 0.039; 3, 0.042; 4, 0.0080; 5, 0.0027.

In addition, we wanted to assess whether the addition of 4′‐thiouridine instigated any altered cytotoxic responses compared with the standard 2′‐MOE uridine. Therefore, apoptosis and necrosis were measured over 72 h following gymnotic delivery of Seq0 or Seq1. For apoptosis, the profiles of both ASOs were very similar, with the luminescence signal remaining constant at earlier timepoints, but increasing with dose at 72 h (Figure 4A,C,E). For necrosis, fluorescent signals increased with dose across all timepoints, with consistently higher signals at 72 h versus 24 h for both ASOs (Figure 4B,D). Interestingly, there is some indication of reduced toxicity of the 4′‐thioribose‐containing Seq1 at the highest doses, with a significant increase in fluorescence at 1000 nM for Seq0 after 24 h (Figure 4F); generally, Seq1 does not cause any obvious increase in toxicity compared with Seq0. In parallel, cells were treated with the same set of ASO doses and the level of *PTEN* quantified (Figure 4G). As expected from the data described above, significant on‐target knockdown was shown at 80 nM; however, the suggestion of increased necrosis at concentrations above 200 nM may explain the increased variability in *PTEN* expression between replicates of 400 nM, 2 μM and 10 μM for Seq0 and the lack of response for 10 μM of Seq1 due to cytotoxicity (Figure 4G).

**FIGURE 5:**
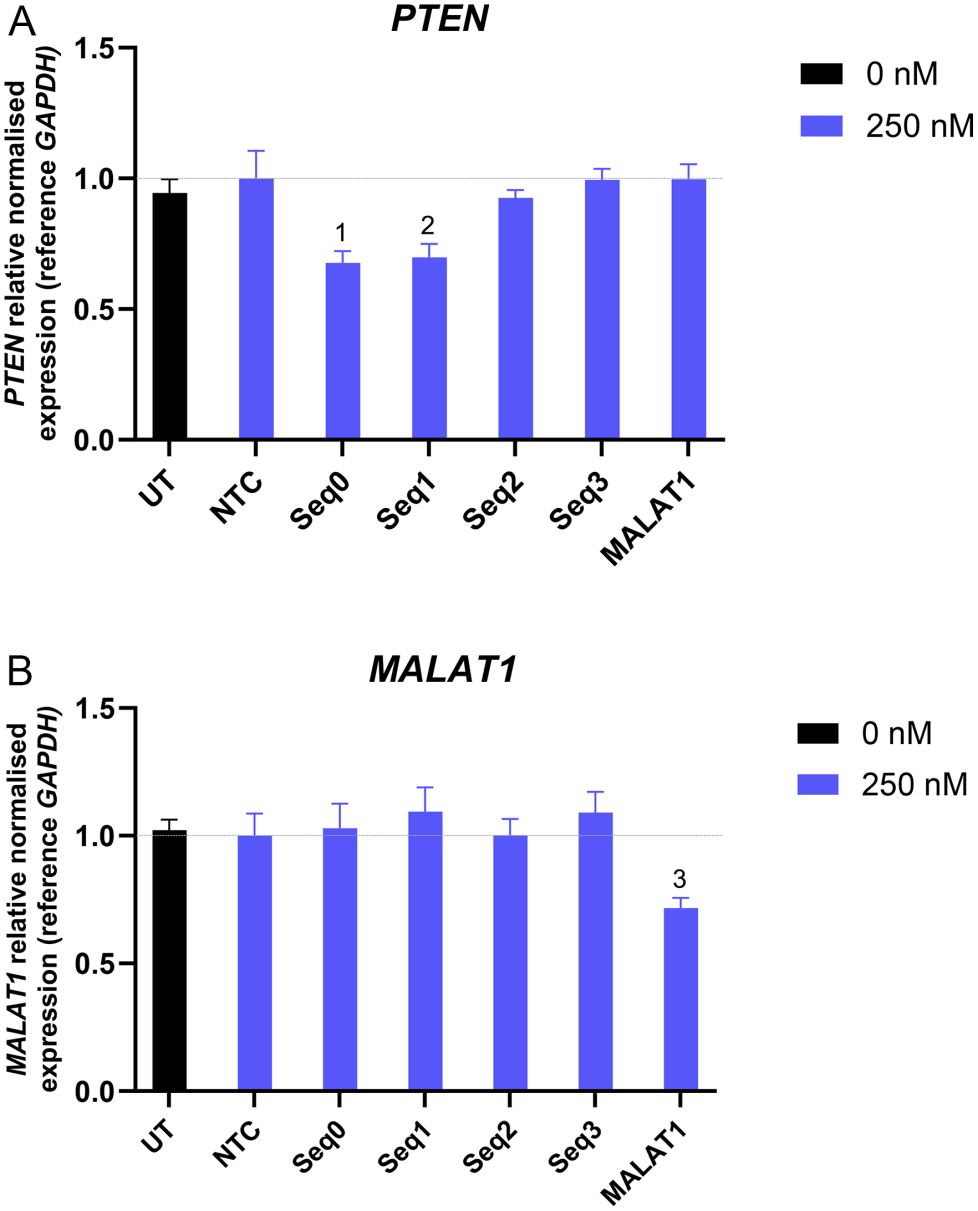
Relative normalised expression of *PTEN* following treatment with Seq0‐Seq3 in HEK293T cells. HEK cells were transfected with the ASOs indicated for 72 h or left untreated (UT). *PTEN* (A) and *MALAT1* (B) expression as measured by qRT‐PCR is shown relative to a non‐targeting control MOE gapmer (NTC) with all samples normalised to the reference gene *GAPDH*. Data are shown +SEM from *N* = 4 biological replicates. Numbers indicate the difference versus the NTC by 1‐way ANOVA with Dunnett’s multiple comparison test. *p*‐values: 1, 0.0049; 2, 0.0087; 3, 0.067.

To confirm the observed ASO activity was not confined to a single cell type, HEK293T cells were transfected with the same set of ASOs alongside a positive control MOE gapmer targeting *MALAT1*. At a single 250 nM dose, a significant knockdown of *PTEN* was observed by approximately 35% using either Seq0 or Seq1 (Figure 5A), while *MALAT1* knockdown was only observed using the *MALAT1* ASO (Figure 5B). Together, these in vitro data indicate that the gapmer Seq 1 with multiple 4′‐thiouridines in the wings has a similar activity and equivalent cytotoxicity profile compared with a standard 2′‐MOE ASO of the same sequence and PS bond content.

### 2.3 A 4′‐Thiouridine‐PS Gapmer ASO Targeting *PTEN* Demonstrates Equivalent Nuclease Resistance in Serum Compared with a Standard 2′‐MOE‐PS Gapmer ASO

**FIGURE 6:**
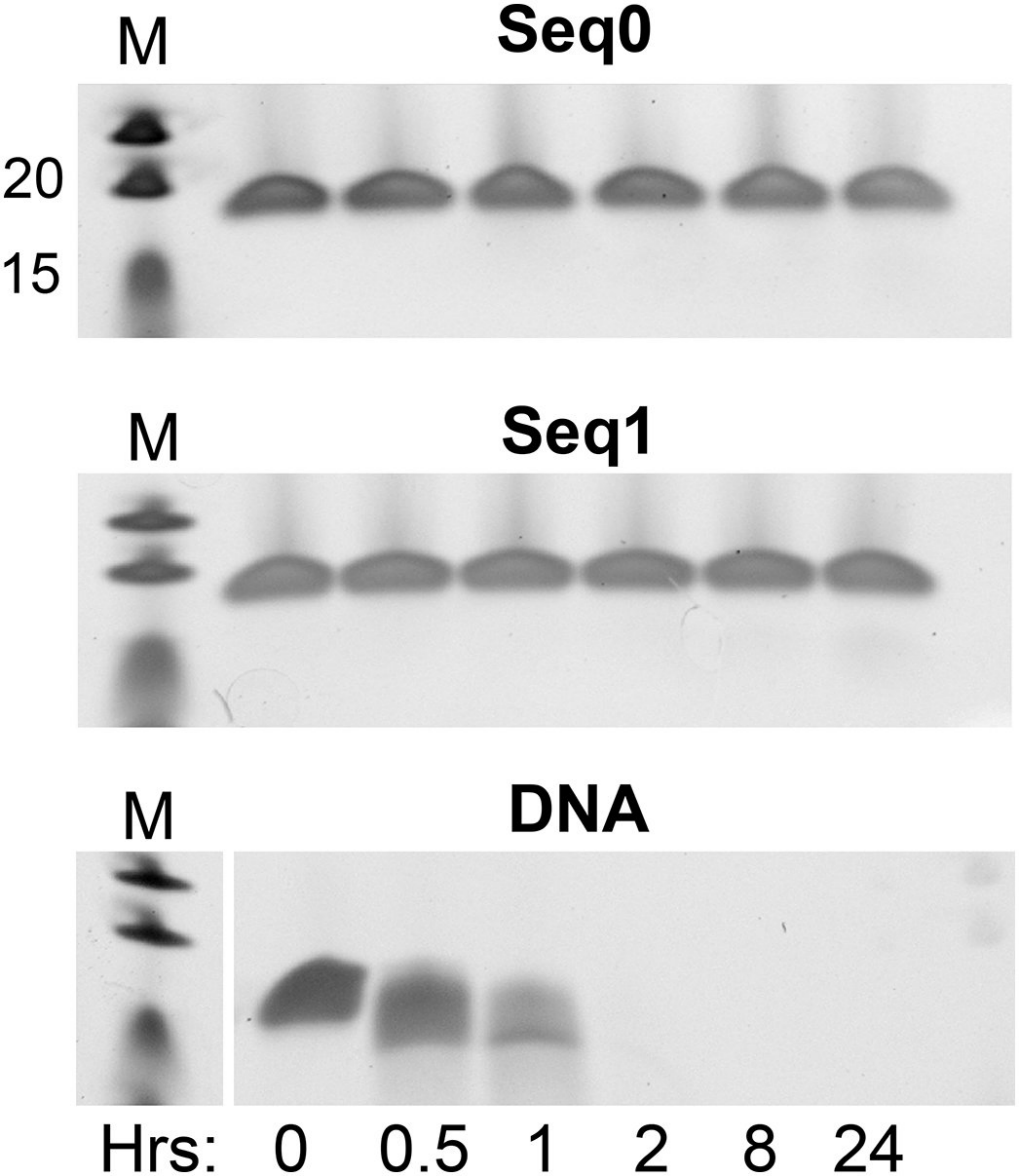
Serum stability of Seq0 and Seq1 ASOs over 24 h. Seq0 and Seq1 gapmer ASOs were incubated for the times indicated in mouse serum at 37 °C. The positive control ASO (DNA) was assayed in parallel and products resolved by gel electrophoresis. The DNA size marker (M) is shown with 20 and 15 nucleotides highlighted.

Beyond on‐target efficacy, an additional desirable feature for clinically viable nucleic acid therapeutics is their relative stability in biological matrices that contain exonucleases. To examine whether the inclusion of 4′‐thiouridine in this gapmer sequence influences nuclease resistance, the Seq0 and Seq1 ASOs were incubated in mouse serum at 37 °C, alongside an unmodified DNA oligonucleotide control. Across a 24 h time course, there was almost no degradation of either Seq0 or Seq1 when visualised by gel electrophoresis, while as expected, the unmodified ASO was completely degraded by 2 h (Figure 6). These data suggest that the 4′‐thiouridine motif inclusion does not have a detrimental effect on nuclease resistance in serum.

## 3 Conclusion

In this study, we demonstrate that a 4′‐thiouridine modification can be incorporated into the flanking regions of 2′‐MOE gapmer ASOs without compromising on‐target efficacy. Through systematic investigation of four oligonucleotide sequences targeting *PTEN*, we establish that 4′‐thiouridine‐modified gapmers containing a phosphorothioate backbone exhibit knockdown activity equivalent to or exceeding that of a standard 2′‐MOE gapmer, under both transfection and gymnotic delivery conditions. Furthermore, the importance of phosphorothioate linkages was demonstrated through observed reduced activity of 4′‐thiouridine‐phosphodiester‐containing sequences, consistent with the essential role of this nucleic acid backbone modification. Importantly, cytotoxicity analyses revealed that 4′‐thiouridine incorporation does not increase cellular toxicity, and this favourable profile, combined with maintained efficacy, suggests that 4′‐thiouridine modification offers an intriguing alternative or complement to established structural modifications within gapmer ASO design.

## Author Contributions

P.L.O. and G.J.M. designed the project. J.E.P., C.M.M.B., A.L.M., J.R., H.L.H., S.B., L.M.C., A.N.C. and P.L.O. performed the experiments and collected and analysed the data. G.J.M., A.N.C. and S.L.L. supervised the academic project. C.M.M.B., P.L.O. and G.J.M. wrote the manuscript. All authors reviewed and commented on the final version.

## Funding

This study was supported by the Medical Research Council (MR/W029324/1, MR/Z000025/1 and MC/PC/20061).

## Conflicts of Interest

The authors declare no conflicts of interest.

## Supporting information

Supplementary Material

## Data Availability

The data that supports the findings of this study are available in the Supporting Information of this article.
