# Supplementary material for: A 4′‐Thioribouridine Modification Mimics 2′‐O‐Methoxyethyl in ASO‐Like 20‐Mers Targeting PTEN Knockdown
Source: Chembiochem. 2026 Sep 27;27(18):e70533. doi: 10.1002/cbic.70533 (PMC13616438; doi:10.1002/cbic.70533)
Supplement: Supplementary file 1 [file CBIC-27-e70533-s001.pdf]

Supporting Information

A 4′-thioribouridine modification mimics 2′-*O*-methoxyethyl in ASO-like 20-mers targeting *PTEN* knockdown

Joanna E. Parkes,[1$] Caecilie M. M. Benckendorff,[2$] Anna L. Malinowska,[1] Jacob Roberts,[2] Harley L. Huynh,[1] Sritama Bose,[1] Loïc M. Cochard,[1] Aisling Ní Cheallaigh,[2] Sarah L. Lovelock,[2] Peter L. Oliver[1] and Gavin J. Miller[2,*]

[1] MRC Nucleic Acid Therapy Accelerator (NATA), Research Complex at Harwell (RCaH), Harwell, Oxford, OX11 0FA, UK.

[2] Manchester Institute of Biotechnology & Department of Chemistry, University of Manchester, Manchester, M1 7DN, UK.

* To whom correspondence should be addressed. Email: gavin.miller@manchester.ac.uk

[$] Equal Contribution

Table of Contents

1. Chemical synthesis of 4′-thiouridine-3′-*O*-phosphoramidite derivative 4 2

1.1 General Experimental 2

1.2 [S(*R*/*S*)]-1-Deoxy-2,3-*O*-isopropylidene-4-*O*-*tert*-butyldiphenylsilyl-*S*-oxo-4-thio-D-ribofuranose (S1) 3

1.3 2′,3′-*O*-Isopropylidene-5′-*O*-*tert*-butyldiphenylsilyl-4′-thiouridine (2)........................4

1.4 2′-*O*-*tert*-butyldimethylsilyl-3′-*O*-[(2-cyanoethoxy)(*N*,*N*-diisopropylamino)phosphino]-5′-*O*-dimethoxytrityl-4′-thiouridine (4) 5

2. Oligonucleotide Synthesis..6

2.1 Solid-Phase Oligonucleotide Synthesis 6

2.2 Deprotection of 2′-OTBS group...7

2.3 DMT removal and purification 7

2.4 Agilent HPLC purification 7

2.5 Oligonucleotide LCMS Data...8

3. *In Vitro* Studies..8

3.1 Cell culture and treatments 8

3.2 RNA extraction and qRT-PCR...8

3.3 Cytotoxicity assay 9

3.4 Serum Stability..8

4. NMR Spectra 10

5. References...17

1. Chemical synthesis of 4′-thiouridine-3′-*O*-phosphoramidite derivative 4

1.1 General Experimental

All chemicals were purchased from Acros Organics, Alfa Aesar, Biosynth, Fisher Scientific, Fluorochem, Sapala Organics, Sigma Aldrich or TCI Chemicals and were used without further purification unless otherwise stated. DMTrCl was recrystalised from hexane prior to use. Anhydrous DMF, MeOH, THF and pyridine were obtained from Sure/Seal™ bottles *via* chemical suppliers. Anhydrous MeCN was obtained by drying over 3 Å molecular sieves and stored in a J Young® flask. All reactions were conducted using anhydrous solvents, under an atmosphere of N_2, unless otherwise stated. Thin layer chromatography (TLC) was performed using pre-coated 0.25 mm 60 F254 silica gel plates (Merck). Visualisation was achieved using UV light (λ = 254 nm), and/or 5% H_2SO_4 in EtOH followed by heating. Flash column chromatography was performed using silica gel, high purity grade, pore size 60 Å, 230-400 mesh particle size, 40-63 μm particle size (Sigma Aldrich). All high-resolution mass spectra were measured at the EPSRC National Mass Spectrometry Facility at Swansea University, UK. NMR spectra were recorded on a Bruker Avance 400 spectrometer. The chemical shift data for ^{1}H and ^{13}C signals are given as δ in units of parts per million (ppm) relative to tetramethylsilane, where δ = 0.00 ppm. The number of protons (n) for a given resonance is indicated by nH. The multiplicity of each signal is indicated by: s (singlet), br s (broad singlet), d (doublet), t (triplet), q (quartet), dd (doublet of doublets), ddd (doublet of doublet of doublets), dt (doublet of triplets), tt (triplet of triplets) or m (multiplet). Coupling constants (*J*) are quoted in Hz and calculated to the nearest 0.1 Hz.

SI Scheme

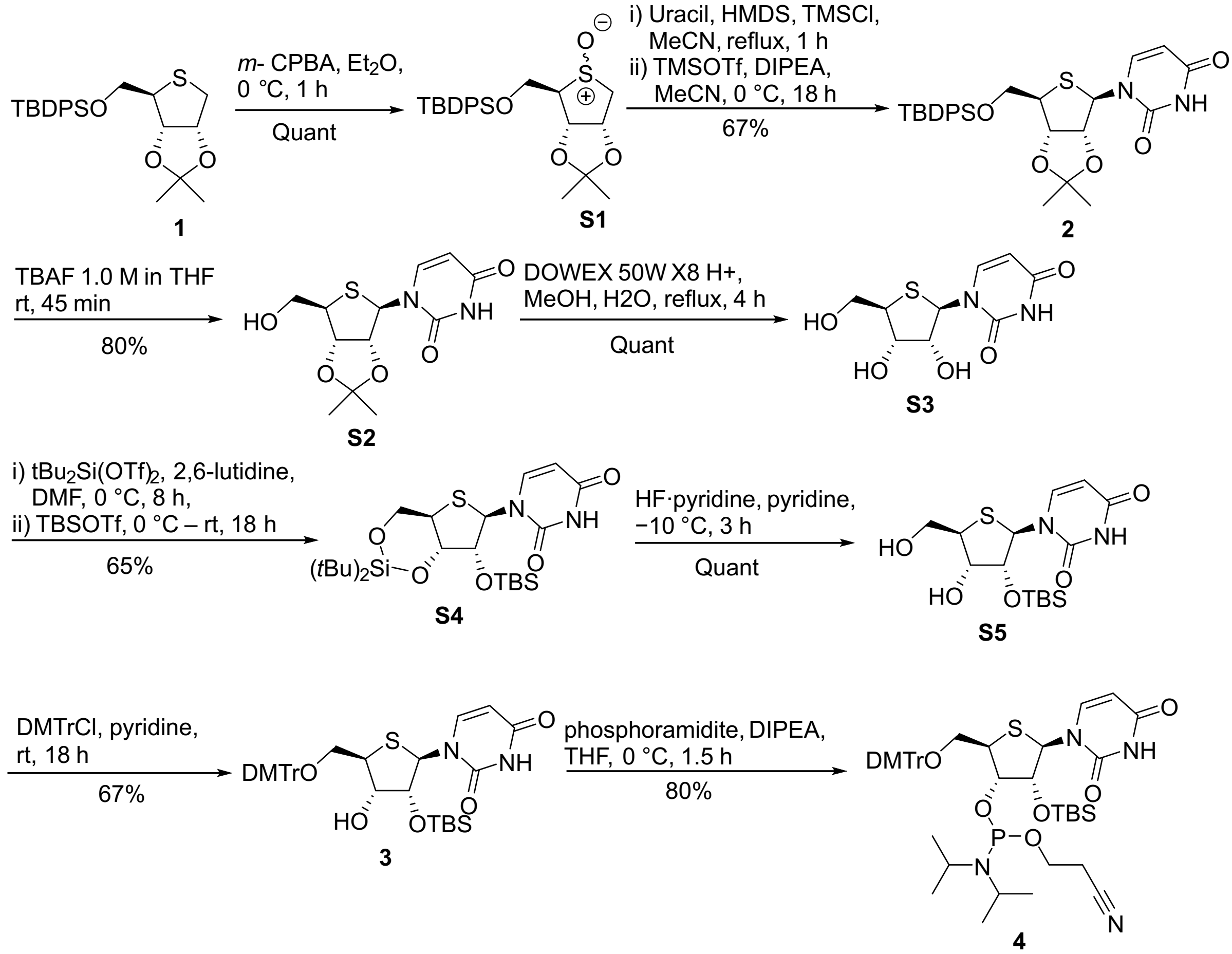

Compounds **S2**, **S3**, **S4**, **S5** and **3** were synthesised according to our previous protocol.[1]

1.2 [S(*R*/*S*)]-1-Deoxy-2,3-*O*-isopropylidene-4-*O*-*tert*-butyldiphenylsilyl-S-oxo-4-thio-D-ribofuranose (S1)

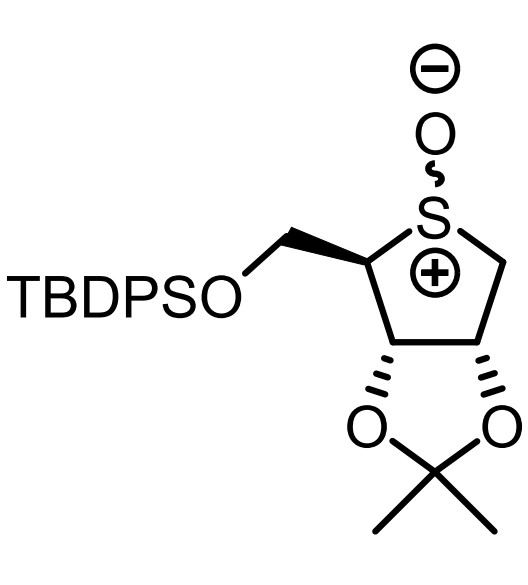

S1

1-deoxy-4-thioribose derivative **1** (2.1 g, 4.9 mmol, 1.0 equiv.) was dissolved in Et_2O (48 mL) and the mixture was cooled to 0 °C. *m*-CPBA (1.2 g, 5.3 mmol, 1.1 equiv.) was added in two portions, and the reaction mixture was stirred at this temperature for 1 h, at which point the TLC analysis (1:1, petroleum ether/EtOAc) showed complete consumption of the starting material to two spots with lower R_f. The reaction was quenched by the addition of sat. $NaHCO_3$ (20 mL) and sat. $Na_2S_2O_3$ (20 mL), and the aqueous layer was extracted with Et_2O (2 x 50 mL). The combined organic phases were washed with H_2O (50 mL), brine (50 mL), dried over $MgSO_4$, filtered and the solvent removed *in vacuo* to give **S1** (2.2 g, 4.9 mmol, quant.), a colourless syrup, and as a 1:0.13 mixture of diastereoisomers. The crude material was used in the next reaction without further purification.

$R_{f(top)}$ = 0.51 (1:1, hexane/EtOAc); $R_{f(bottom)}$ = 0.34 (1:1 hexane/EtOAc); NMR analysis: Observed for both diastereoisomers: ^{1}H NMR (400 MHz, $CDCl_3$): δ 7.47 – 7.38 (6.88H, m, ArH), 3.45 – 3.40 (1.17H, m, $H_{1a(a)}$, $H_{1a(b)}$), 3.30 – 3.26 (1.15H, m, $H_{1b(a)}$, $H_{4(b)}$).

Major diastereoisomer: ^{1}H NMR (400 MHz, $CDCl_3$): δ 7.73 – 7.68 (4H, m, ArH), 5.17 (1H, td, $^{3}J_{H2\text{-}H1a/H3}$ = 6.4 Hz, $^{3}J_{H2\text{-}H1b}$ = 4.0 Hz, H_2), 4.83 (1H, dd, $^{3}J_{H3}$ = 6.4 Hz, $^{3}J_{H3}$ = 5.2 Hz, H_3), 4.18 (1H, dd, $^{2}J_{H5a\text{-}H5b}$ = 11.2 Hz, $^{3}J_{H5a\text{-}H4}$ = 4.3 Hz, H_{5a}), 3.20 (1H, dd, $^{2}J_{H1b}$ = 14.0 Hz, $^{3}J_{H1b\text{-}H2}$ = 4.0 Hz, H_{1b}), 1.44 (3H, s, CH_3), 1.28 (3H, s, CH_3), 1.08 (9H, s, [$C(CH_3)_3$]); ^{13}C NMR (101 MHz, $CDCl_3$): δ 135.83 (ArC), 135.82 (ArC), 132.9 (ArC), 132.7 (ArC), 130.10 (ArC), 130.05 (ArC), 127.99 (ArC), 127.98 (ArC), 112.9 [$\mathit{C}(CH_3)_2$], 82.1 (C_3), 80.1 (C_2), 67.6 (C_4), 58.0 (C_5), 56.1 (C_1), 27.3 [$C(\mathit{C}H_3)_2$], 26.9 [$C(\mathit{C}H_3)_3$], 24.7 [$C(\mathit{C}H_3)_2$], 19.32 [$\mathit{C}(CH_3)_3$].

Minor diastereoisomer: ^{1}H NMR (400 MHz, $CDCl_3$): δ 7.62 – 7.53 (0.73H, m, ArH), 5.23 (0.14H, td, $^{3}J_{H2\text{-}H1a/H3}$ = 5.9 Hz, $^{3}J_{H2\text{-}H1b}$ = 1.7 Hz, H_2), 4.99 (0.13H, dd, $^{3}J_{H3\text{-}H2}$ = 5.9 Hz, $^{3}J_{H3\text{-}H4}$ = 1.7 Hz, H_3), 4.11 (0.17H, ov. dd, $^{2}J_{H5a\text{-}H5b}$ = 11.2 Hz, $^{3}J_{H5a\text{-}H4}$ = 2.8 Hz, H_{5a}), 3.85 (0.14H, dd, $^{2}J_{H5b\text{-}H5a}$ = 11.2 Hz, $^{3}J_{H5b\text{-}H4}$ = 3.5 Hz, H_{5b}), 3.61 (0.15H, br. s, H_4), 1.58 (0.36H, s, CH_3), 1.35 (0.35H, s, CH_3), 1.04 (0.78H, s, [$C(C\mathit{H})_3$]); ^{13}C NMR (101 MHz, $CDCl_3$): δ 135.7 (ArC), 135.6 (ArC), 132.0 (ArC), 131.9 (ArC), 130.44 (ArC), 130.37 (ArC), 128.20 (ArC), 128.17 (ArC), 112.6 [$\mathit{C}(CH_3)_3$], 86.7 (C_3), 85.0 (C_2), 75.0 (C_4), 62.0 (C_5), 58.6 (C_1), 27.0 [$C(\mathit{C}H_3)_3$], 26.9 [$C(\mathit{C}H_3)_2$], 24.6 [$C(\mathit{C}H_3)_2$], 19.26 [$\mathit{C}(CH_3)_3$]. These data were in good agreement with literature.[2]

1.3 2′,3′-*O*-Isopropylidene-5′-*O*-*tert*-butyldiphenylsilyl-4′-thiouridine (2)

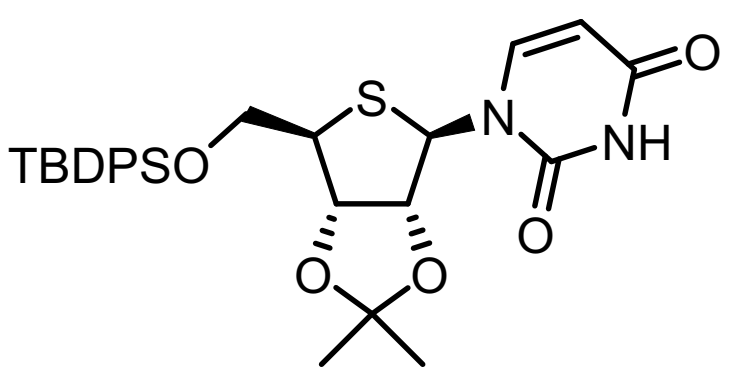

2

Uracil (1.1 g, 9.7 mmol, 2.0 equiv.) was charged to an oven-dried three necked round bottom flask equipped with a magnetic stirrer. The round bottom flask was evacuated and refilled with N_2 x 3. MeCN (17 mL) was added, followed by HMDS (5.5 mL, 29.1 mmol, 6.0 equiv.) and TMSCl (400 µL, 2.9 mmol, 0.6 equiv.). The suspension was heated to reflux for approx. 1 h, until a clear solution was obtained. The reaction mixture was cooled to rt, and **S1** (2.2 g, 4.9 mmol, 1.0 equiv.) in MeCN (83 mL) was added. The reaction mixture was cooled to 0 °C, and DIPEA (5.1 mL, 29.1 mmol, 6.0 equiv.) was added, followed by TMSOTf (5.3 mL, 29.1 mmol, 6.0 equiv.) dropwise. The reaction mixture was allowed to warm slowly to rt and stirred for a further 16 h, until TLC analysis (1:1, petroleum ether/EtOAc) showed complete consumption of the starting material from two spots with lower R_f to two spots with higher R_f. The reaction mixture was concentrated *in vacuo* and partitioned between H_2O (200 mL) and EtOAc (100 mL). The aqueous phase was washed with EtOAc (3 x 200 mL), and the combined organic phase was washed with brine (200 mL), dried over $MgSO_4$, filtered and the solvent removed *in vacuo*. The crude material, a 1:5 α/β mixture of anomers, was purified *via* flash

column chromatography in silica gel (10 – 50% EtOAc/petroleum ether) to give **2** (1.8 g, 3.3 mmol, 67%) as a white foam.

R_f = 0.5 (1:1, hexane/EtOAc); ^{1}H NMR (400 MHz, $CDCl_3$): δ 7.68 – 7.63 (5H, m, ArH, H_6), 7.48 – 7.44 (2H, m, ArH), 7.42 – 7.38 (4H, m, ArH), 6.06 (1H, d, $^3J_{H1'-H2'}$ = 3.1 Hz, $H_{1'}$), 5.47 (1H, d, $^3J_{H5-H6}$ = 8.1 Hz, H_5), 4.74 (1H, dd, $^3J_{H3'-H2'}$ = 5.6 Hz, $^3J_{H3'-H4'}$ = 2.8 Hz, $H_{3'}$), 4.66 (1H, dd, $^3J_{H2'-H3'}$ = 5.6 Hz, $^3J_{H2'-H1'}$ = 3.1 Hz, $H_{2'}$), 3.89 (2H, app. d, $^3J_{H5'a/H5'b-H4'}$ = 5.7 Hz, $H_{5'a}$, $H_{5'b}$), 3.76 (1H, app. td, $^3J_{H4'-H5'a/H5'b}$ = 5.6 Hz, $^3J_{H4'-H3'}$ = 2.9 Hz, $H_{4'}$), 1.58 (3H, s, CH_3), 1.31 (3H, s, CH_3), 1.10 (9H, s, 3 x CH_3); ^{13}C NMR (101 MHz, $CDCl_3$): δ 162.3 (C_2, C=O), 150.0 (C_4, C=O), 141.1 (C_6), 135.6 (ArC), 135.5 (ArC), 132.9 (ArC), 132.5 (ArC), 130.1 (ArC), 127.9 (ArC), 112.6 [$C(CH_3)_2$], 102.9 (C_5), 88.9 ($C_{2'}$), 84.1 ($C_{3'}$), 68.2 ($C_{1'}$), 65.1 ($C_{5'}$), 55.6 ($C_{4'}$), 27.5 [$C(CH_3)_2$], 27.0 [$C(CH_3)_3$], 25.3 [$C(CH_3)_2$], 19.4 [$C(CH_3)_3$]; HRMS (ESI): *m/z* Calculated for $C_{28}H_{34}N_2O_5SSi$ $[M+H]^+$ 539.2036, found 539.2043. This data is in good agreement with literature.[1]

1.4 2′-*O*-*tert*-butyldimethylsilyl-3′-*O*-[(2-cyanoethoxy)(N,N-diisopropylamino)phosphino]-5′-*O*-dimethoxytrityl-4′-thiouridine (4)

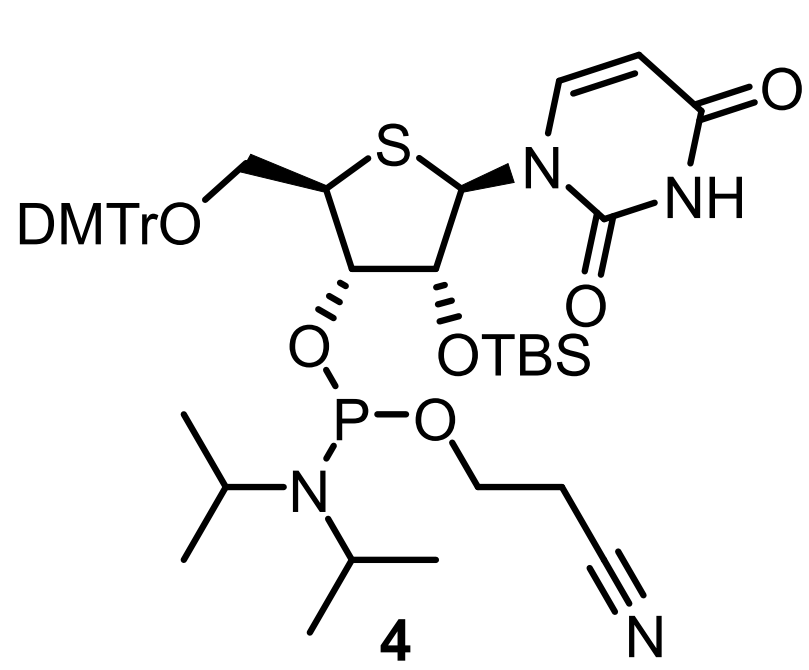

4

3′-Hydroxyl **3** (400 mg, 0.59 mmol, 1.0 equiv.) was charged to an oven dried three-necked round-bottom flask equipped with a magnetic stirrer bar. The reaction vessel was evacuated and refilled with N_2 three times. THF (4.7 mL) was added, followed by DIPEA (618 μL, 3.6 mmol, 6.0 equiv.). The reaction mixture was cooled to 0 °C, and 2-cyanoethyl diisopropylchlorophosphoramidite (317 μL, 1.2 mmol, 2.0 equiv.) was added dropwise. The reaction mixture was allowed to warm to rt and stirred for a further 16 h, at which point TLC analysis (1:1, hexane/EtOAc) showed complete consumption of the starting material to two spots with higher R_f. The reaction mixture was concentrated *in vacuo*, and the residue was purified *via* flash column chromatography on silica gel neutralised with Et_3N (0 – 40% EtOAc/hexane +1% Et_3N) to give 3′-*O*-phosphoramidite **4** (412 mg, 0.47 mmol, 80%) a 1:0.7 mixture of diastereoisomers, as a white foam.

The P diastereoisomers were not separated and the following assignments are derived from the constituent mixture: R_f = 0.38 and 0.43 (1:1, hexane/EtOAc); ^{1}H NMR (400 MHz, $CDCl_3$): δ 8.03 (1H, d, $^3J_{H6-H5}$ = 8.2 Hz, H_6), 7.89 (1H, d, $^3J_{H6-H5}$ = 8.2 Hz, H_6), 7.48 – 7.42 (4H, m, ArH), 7.37 – 7.24 (16H, m, ArH), 6.86 – 6.82 (8H, m, ArH), 5.93 (1H, d, $^3J_{H1'-H2'}$ = 5.2 Hz, $H_{1'}$), 5.86 (1H, d, $^3J_{H1'-H2'}$ = 4.2 Hz, $H_{1'}$), 5.52 (1H, d, $^3J_{H5-H6}$ = 8.2 Hz, H_5), 5.49 (1H, d, $^3J_{H5-H6}$ = 8.2 Hz, H_5), 4.27 (1H, dd, $^3J_{H2'-P}$ = 5.2 Hz, $^3J_{H2'-H3'}$ = 3.2 Hz, $H_{2'}$), 4.24 (1H, dd, $^3J_{H2'-P}$ = 4.3 Hz, $^3J_{H2'-H3'}$ = 3.1 Hz, $H_{2'}$), 4.20 – 4.11 (2H, m, 2 x $H_{3'}$), 3.93 – 3.87 (1H, m, CH_2CH_2CN), 3.80 (12H, ov. s, 4 x OCH_3), 3.78 – 3.68 (3H, m, $H_{4'}$, 2 x CH_2CH_2CN), 3.65 – 3.45 (8H, m, $H_{4'}$, $H_{5'a}$, $H_{5'b}$,

CH_2CH_2CN, 4 x [$CH(CH_2)_3$]), 3.45 – 3.38 (2H, m, $H_{5'a}$, $H_{5'b}$), 2.62 (2H, t, $^3J_{H-H}$ = 6.1 Hz, CH_2CH_2CN), 2.44 (2H, td, $^3J_{H-H}$ = 6.3 Hz, $^3J_{H-P}$ = 1.9 Hz, CH_2CH_2CN), 1.16 (6H, d, $^3J_{H-H}$ = 5.4 Hz, [$CH(CH_3)_2$]), 1.14 (6H, d, $^3J_{H-H}$ = 5.5 Hz, [$CH(CH_3)_2$]), 1.12 (6H, d, $^3J_{H-H}$ = 6.7 Hz, [$CH(CH_3)_2$]), 1.04 (6H, d, $^3J_{H-H}$ = 6.8 Hz, [$CH(CH_3)_2$]), 0.88 (18H, s, [$C(CH_3)_3$]), 0.11 (3H, s, CH_3), 0.09 (6H, ov. s, 2 x CH_3), 0.08 (3H, s, CH_3); ^{13}C NMR (101 MHz, $CDCl_3$): δ 162.80 (C_4, C=O), 162.76 (C_4, C=O), 158.9 (ArC), 158.8 (ArC), 150.7 (C_2, C=O), 150.6 (C_2, C=O), 144.6 (ArC), 144.5 (ArC), 141.7 (C_6), 141.4 (C_6), 135.6 (ArC), 135.53 (ArC), 135.47 (ArC), 135.4 (ArC), 130.4 (ArC), 130.33 (ArC), 130.30 (ArC), 130.27 (ArC), 128.34 (ArC), 128.26 (ArC), 128.0 (ArC), 127.9 (ArC), 127.1 (ArC), 117.7 (CH_2CH_2CN), 117.4 (CH_2CH_2CN), 113.20 (ArC), 113.17 (ArC), 113.15 (ArC), 102.4 (C_5), 102.1 (C_5), 87.11 [$C(Ph)(Ph\text{-}OCH_3)_2$], 87.07 [$C(Ph)(Ph\text{-}OCH_3)_2$], 78.5 – 78.2 (m, $C_{2'}$), 75.6 – 75.0 (m, $C_{3'}$), 65.2 ($C_{1'}$), 64.7 ($C_{1'}$), 63.7 ($C_{5'}$), 63.3 ($C_{5'}$), 58.2 (d, $^2J_{C-P}$ = 18.6 Hz, CH_2CH_2CN), 57.8 (d, $^2J_{C-P}$ = 19.8 Hz, CH_2CH_2CN), 55.28 (OCH_3), 55.25 (OCH_3), 49.5 ($C_{4'}$), 43.3 (d, $^2J_{C-P}$ = 12.7 Hz, [$CH(CH_3)_2$]), 43.1 (d, $^2J_{C-P}$ = 12.6 Hz, [$CH(CH_3)_2$]), 25.7 [$C(CH_3)_3$], 25.6 [$C(CH_3)_3$], 24.7 – 24.5 (m, [$CH(CH_3)_2$]), 20.5 (d, $^3J_{C-P}$ = 6.3 Hz, CH_2CH_2CN), 20.3 (d, J = 6.7 Hz, CH_2CH_2CN), 17.93 [$C(CH_3)_3$], 17.91 [$C(CH_3)_3$], −4.71 (CH_3), −4.73 (CH_3), −4.75 (CH_3), −4.79 (CH_3); $^{31}P\{^1H\}$ NMR (162 MHz, $CDCl_3$): δ 149.8 (s), 149.7 (s); HRMS (NSI): *m*/*z* Calculated for $C_{45}H_{62}N_4O_8PSSi$ $[M+H]^+$ 877.3790, found 877.3809. These data are in good agreement with literature.[1]

2. Oligonucleotide Synthesis

2.1 Solid-Phase Oligonucleotide Synthesis

Oligonucleotides were synthesized at a 1 µmol scale with the MM12 synthesizer (LGC Biosearch Technologies) by the standard phosphoramidite method, using 1000 Å Universal Controlled Pore Glass (CPG) (loading 47.7 µmol/g, LGC Biosearch Technologies). Phosphoramidites were prepared as 0.1 M solutions in anhydrous MeCN (LGC Biosearch Technologies). Solvents were moisture controlled with less than 30 ppm water content. A 0.25 M solution of 5-ethylthio-1*H*-tetrazole in anhydrous MeCN (LGC Biosearch Technologies) was used as an activator. Failed sequences were capped with a 1:1 mixture of Capping Mix A (THF/lutidine/Ac_2O, 8:1:1, *v*/*v*/*v*, LGC Biosearch Technologies) and Capping Mix B (16% *N*-methylimidazole in THF, LGC Biosearch Technologies). The oxidizing step was performed with 0.02 M iodine in THF/pyridine/H_2O (7:2:1, *v*/*v*/*v*, LGC Biosearch Technologies). After the synthesis, CPG was treated with 32% aqueous ammonia solution (400 µL) and was shaken (800 rpm, Eppendorf ThermoMixer C) for 14 hours at 37 ºC. The samples were then left to cool to room temperature, filtered, and freeze-dried.

2.2 Deprotection of 2′-OTBS group

Samples were dissolved in anhydrous DMSO (250 µL). $Et_3N{\cdot}3HF$ (125 µL) was added and the samples were incubated at 37 ºC overnight. The reaction was quenched with trimethylethoxysilane (350 µL, 10 min, room temperature). Et_2O (350 µL, 10 min, room temperature) was then added and the mixture was vortexed and centrifuged. The supernatant was carefully removed. Samples were dissolved in 200 µL of H_2O, filtered on ULTRAFREE-MC DURA 0.22 µm filters. Analysis was carried out by LCMS (Waters LCMS system with Acquity QDa detector, ACQUITY PREMIER Oligonucleotide BEH C18 column (130 Å; 2.1 x 50 mm, 1.7 µm; Waters) at 65 °C with a flow rate of 0.3 mL/min. Eluent A: 7 mM Et_3N, 80 mM HFIP in H_2O; eluent B: 3.5 mM Et_3N, 40 mM HFIP in 50% MeCN; gradient 5 - 30% B in 8 min). Samples were run in negative mode (ESI^-) and analysed as *m/z* $[M]^-$ or $[M-H]^-$. The purity of the oligonucleotides was auto measured using the integration function on MassLynx software V4.2 (Waters).

2.3 DMTr removal and purification

Samples were dissolved in 0.5 mL of NanoPure H_2O. 0.5 mL of 100 mg/mL NaCl solution was added to the oligonucleotides for a final volume of 1.0 mL. 50 mg cartridges were placed in the female Luer ports of the manifold. Collection tubes were placed in the rack below the output guides. The vacuum was turned on and the pressure adjusted to ~7mm Hg using the vacuum control valve. Cartridges were conditioned using 0.5 mL of MeCN followed by 1.0 mL of 2 M TEAA. The crude oligonucleotides were applied to the cartridges. The cartridges were washed with 1.0 mL of salt wash solution (12% MeCN in 100 mg/mL NaCl). This rinses away the remainder of the failure sequences from the cartridge. Trityl removal step: 2 x 1 mL of 4% TFA were added to the cartridges; the acid was allowed to flow slowly through the cartridge with a targeted contact time of 3-4 minutes. The cartridges were washed with 1.0 mL of deionised H_2O. The appropriate sample tube was placed into the manifold. The purified oligonucleotides were eluted using 2 x 1 mL 50% MeCN in H_2O containing 0.5% NH_4OH and freeze-dried.

2.4 Agilent HPLC purification

Method: Mobile phase A: 0.1 M TEAA in deionised H_2O/Mobile phase B: 0.1 M TEAA in 50% MeCN. Flow: 1 mL/min / Column temp: 65ºC / Gradient: 25-40% B in 20 min or 35-50% B in 20 min. Column: XBridge Premier Oligo BEH C18, 130 Å, 2.5 µm, 4.6 x 150 m (Waters). Fractions with the same retention time were combined, dried and injected on the LCMS QDa (conditions as above). For desalting, samples containing the pure products were desalted on Glen Gel-Pak™ 0.2 Desalting Column (catalogue no 61-5002-50, Glen Research) following

the manufacturer's protocol. Eluted samples were lyophilised, dissolved in H_2O and injected on the LCMS. Samples were quantified on the NanoDrop One and freeze-dried.

2.5 Oligonucleotide LCMS Data

Sequence Number	Mass Calculated	Mass Found $[M-H]^-/[M]^-$	Purity
Seq 0	7147.07	7145.85	90%
Seq 1	6754.79	6755.25	90%
Seq 2	6610.16	6610.50	75%
Seq 3	6449.46	6447.75	82%

3. *In Vitro* Studies

3.1 Cell culture and treatments

HeLa cells were cultured in Dulbecco′s Modified Eagle Medium with 10% foetal bovine serum and 1% Penicillin-Streptomycin. For treatments, cells were seeded at 12,000 cells/well overnight and treated with ASOs at various concentrations for 24 or 72 hours with four replicates per treatment group. Transfection was performed using 0.72 μL Lipofectamine RNAiMAX per well complexed with ASOs in Opti-MEM reduced serum media (Fisher Scientific) before addition to wells. Gymnosis was performed by replacing media in the wells with ASOs diluted in growth media.

3.2 RNA extraction and qRT-PCR

RNA was extracted from HeLa and HEK293T cells using the MagMAX miRVana Total RNA Isolation kit on the Kingfisher Flex System (Thermo Fisher Scientific). Reverse transcription was performed using the High Capacity cDNA conversion kit with RNase inhibitor (Fisher Scientific). qRT-PCR was performed with technical triplicates using Fast SYBR Green Mastermix (Fisher Scientific) with PCR primers for *PTEN* (Forward: 5′-AATGGCTAAGTGAAGATGACAATCAT Reverse: 5′-TGCACATATCATTACACCAGTTCGT), *MALAT1* (Forward: 5'-GCAGGCGTTGTGCGTAGAG Reverse: 5'-TTGCCGACCTCACGGATT) and *GAPDH* as the reference gene (Forward: 5′-GTCTCCTCTGACTTCAACAGCG Reverse: 5′-ACCACCCTGTTGCTGTAGCCAA). Samples were run on the Bio-Rad CFX Opus 384 Real-Time PCR System and results were analysed using CFX Maestro software v2.3 and GraphPad Prism v10.5.0.

3.3 Cytotoxicity assay

Cells were plated as above but in solid white nunclon delta-treated 96-well plates (Thermo Scientific). Immediately following gymnotic delivery of ASOs (described above) the RealTime-Glo Annexin V Apoptosis and Necrosis Assay Detection Reagent was added as recommended by the manufacturer (Promega). Luminescence and fluorescence signals were measured at 0, 2, 4, 24, 48 and 72 hours to determine levels of apoptosis and necrosis, respectively, using the following settings on a Clariostar Plus platereader (BMG Labtech, software version 5.70 R2): Settings for luminescence: top optic with no filters, enhanced dynamic range, measurement interval 1.0s and focal height 11mm. Settings for fluorescence: top optic with excitation 482-16, Diochroic 500, Emission 530-40, 0.2s settling time, 40 flashes, enhanced dynamic range and auto focus for the first measurement to set the focal height. The background signal (no-cell controls) was subtracted from test wells and the normalised data used for analysis in GraphPad Prism v10.5.0.

3.4 Serum stability

Aliquots (10 μl of a 10 μM stock) of Seq0 and Seq1 ASOs were mixed with 90 μl of undiluted mouse serum (Merck) and incubated at 37°C. One sample was taken immediately, followed by 30 minutes, 1 hour, 2 hours, 8 hours and 24 hours. Each sample was loaded in 2 x RNA Loading Buffer (Thermo Scientific) onto a 15% TBE-Urea Gel (Thermo Scientific) and run in 1 x TBE buffer for 90 minutes at 125 V. Gels were stained in 1:10 000 SYBR Gold Nucleic Acid Stain (Thermo Scientific) for 10 minutes in 1 x TBE before imaging by UV translumination in a ChemiDoc Imaging System (BioRad).

4. NMR Spectra

[S(*R*/*S*)]-1-Deoxy-2,3-*O*-isopropylidene-4-*O*-*tert*-butyldiphenylsilyl-*S*-oxo-4-thio-D-ribofuranose (2)

^{1}H NMR (400 MHz, $CDCl_3$)

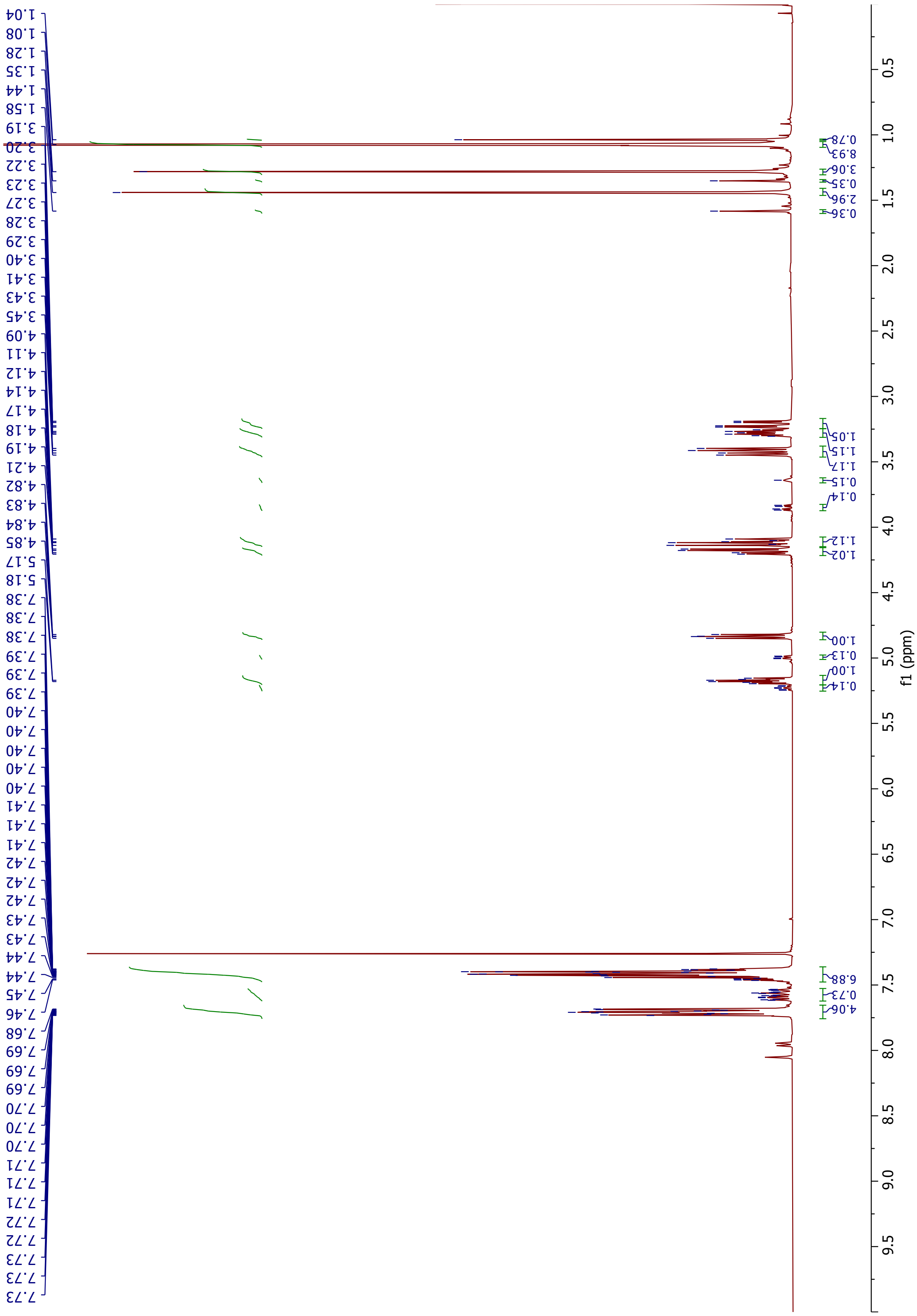

^{13}C NMR (101 MHz, $CDCl_3$)

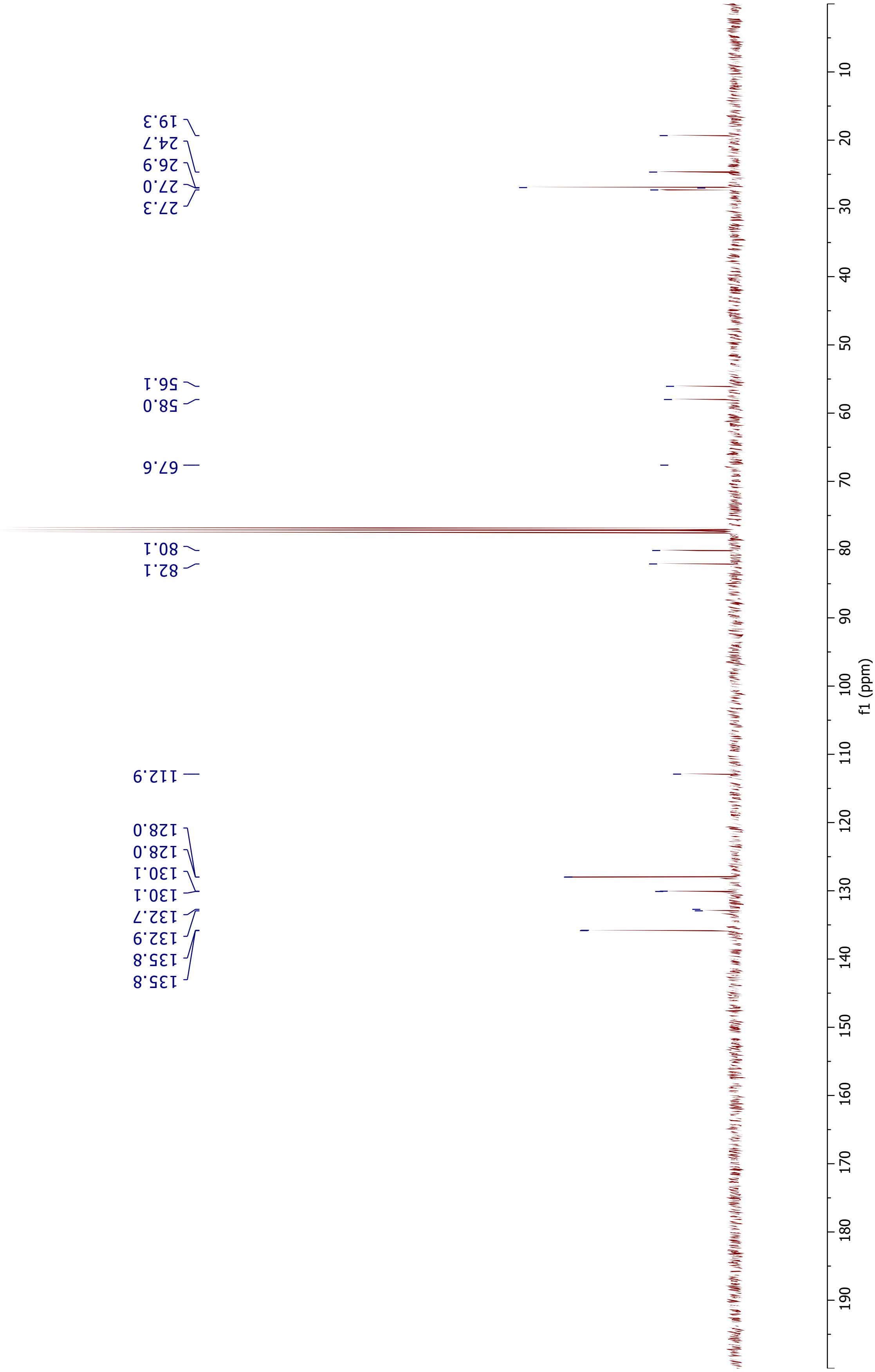

2′,3′-*O*-Isopropylidene-5′-*O*-*tert*-butyldiphenylsilyl-4′-thiouridine (2)

^{1}H NMR (400 MHz, $CDCl_3$)

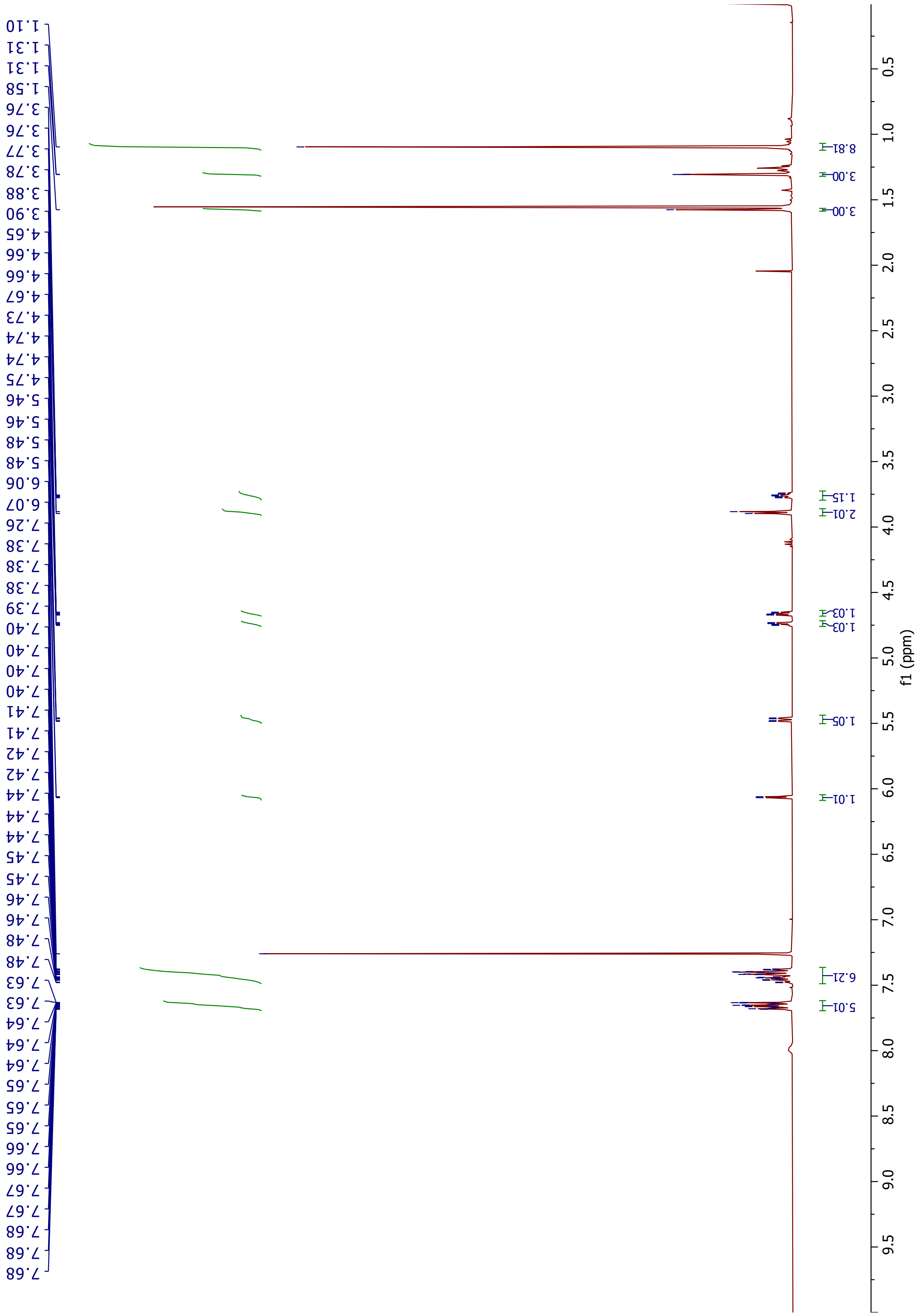

^{13}C NMR (101 MHz, $CDCl_3$)

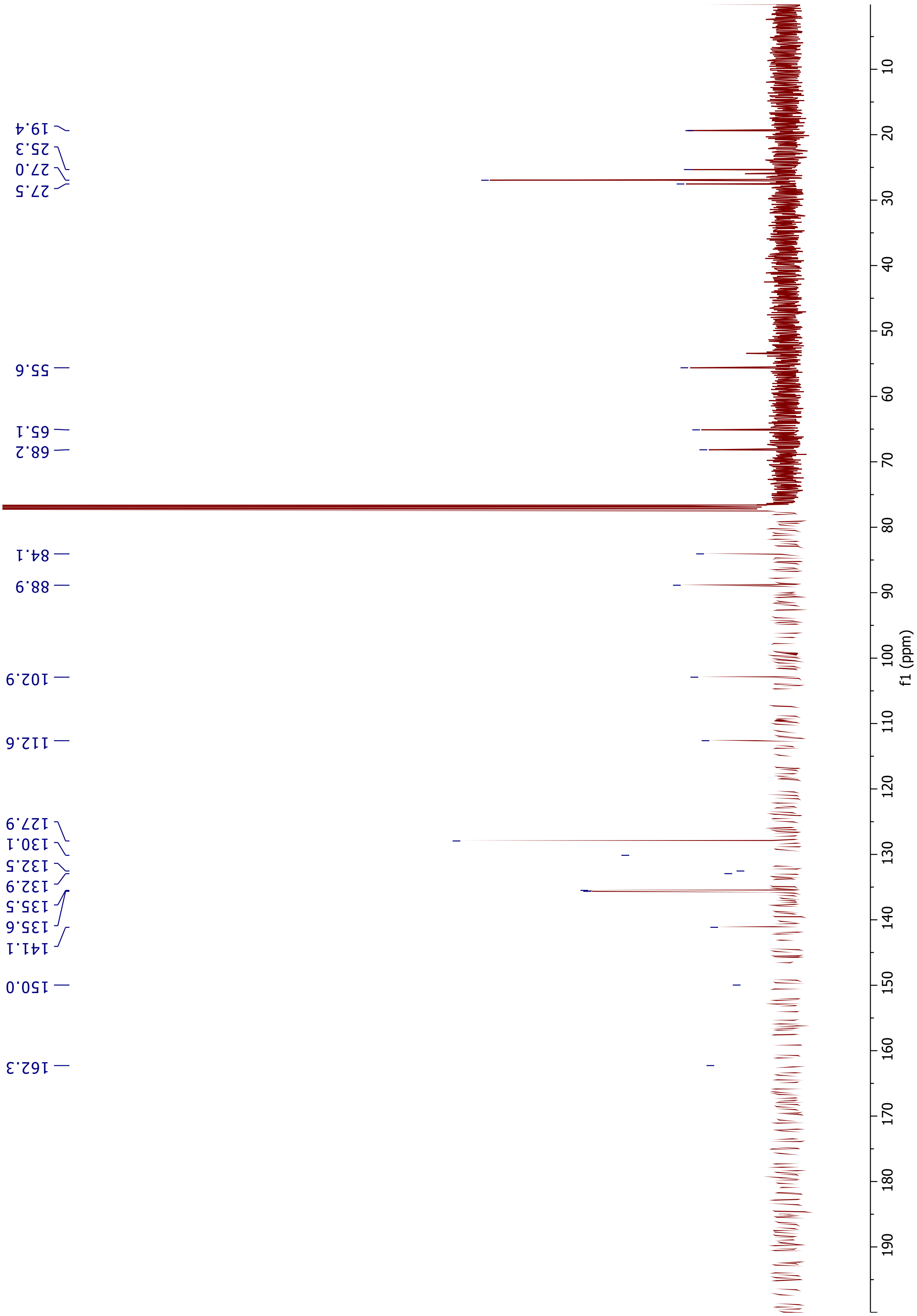

2′-*O*-*tert*-butyldimethylsilyl-3′-*O*-[(2-cyanoethoxy)(*N*,*N*-diisopropylamino)phosphino]-5′-*O*-dimethoxytrityl-4′-thiouridine (4)

^{1}H NMR (400 MHz, $CDCl_3$)

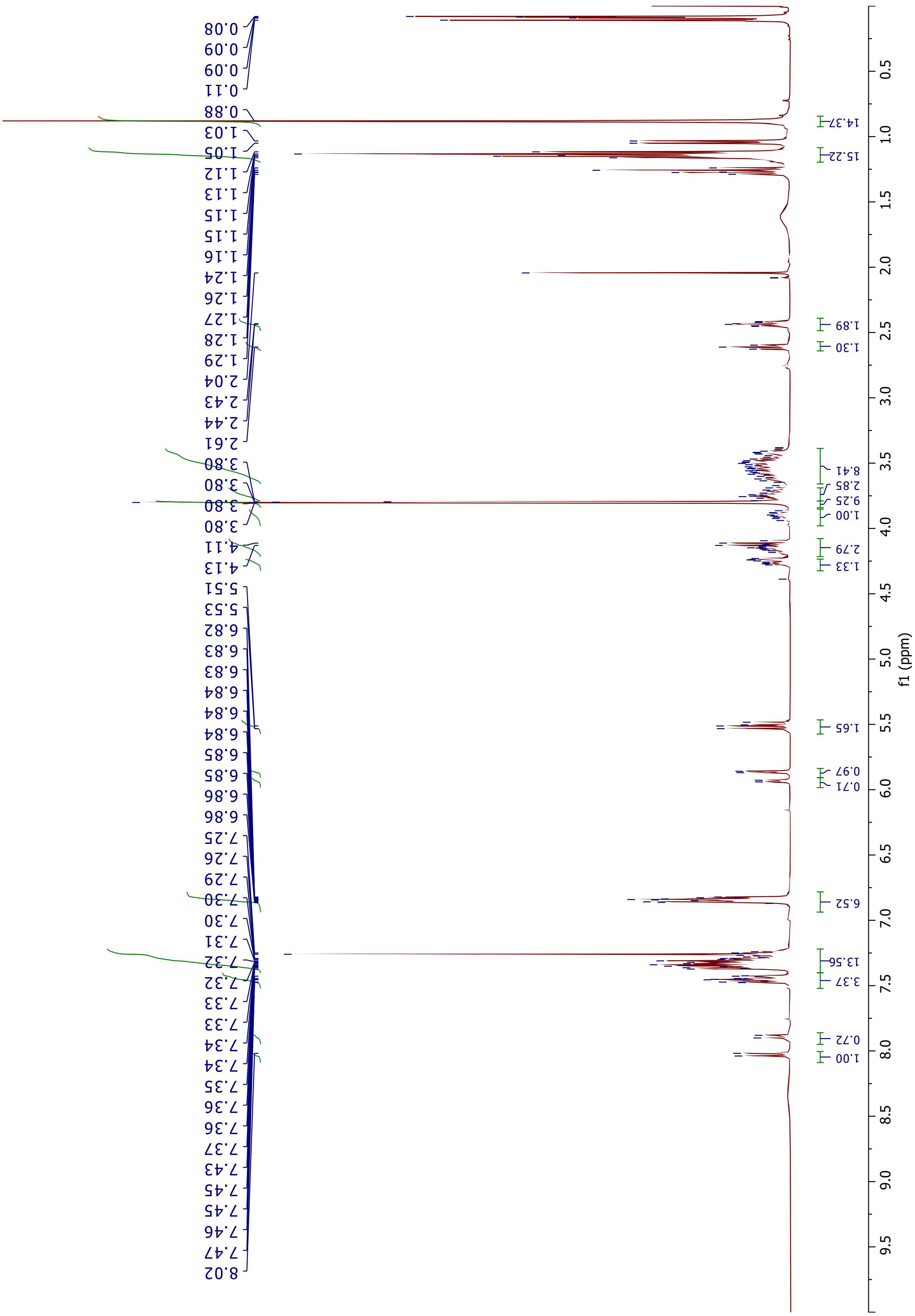

^{13}C NMR (101 MHz, $CDCl_3$)

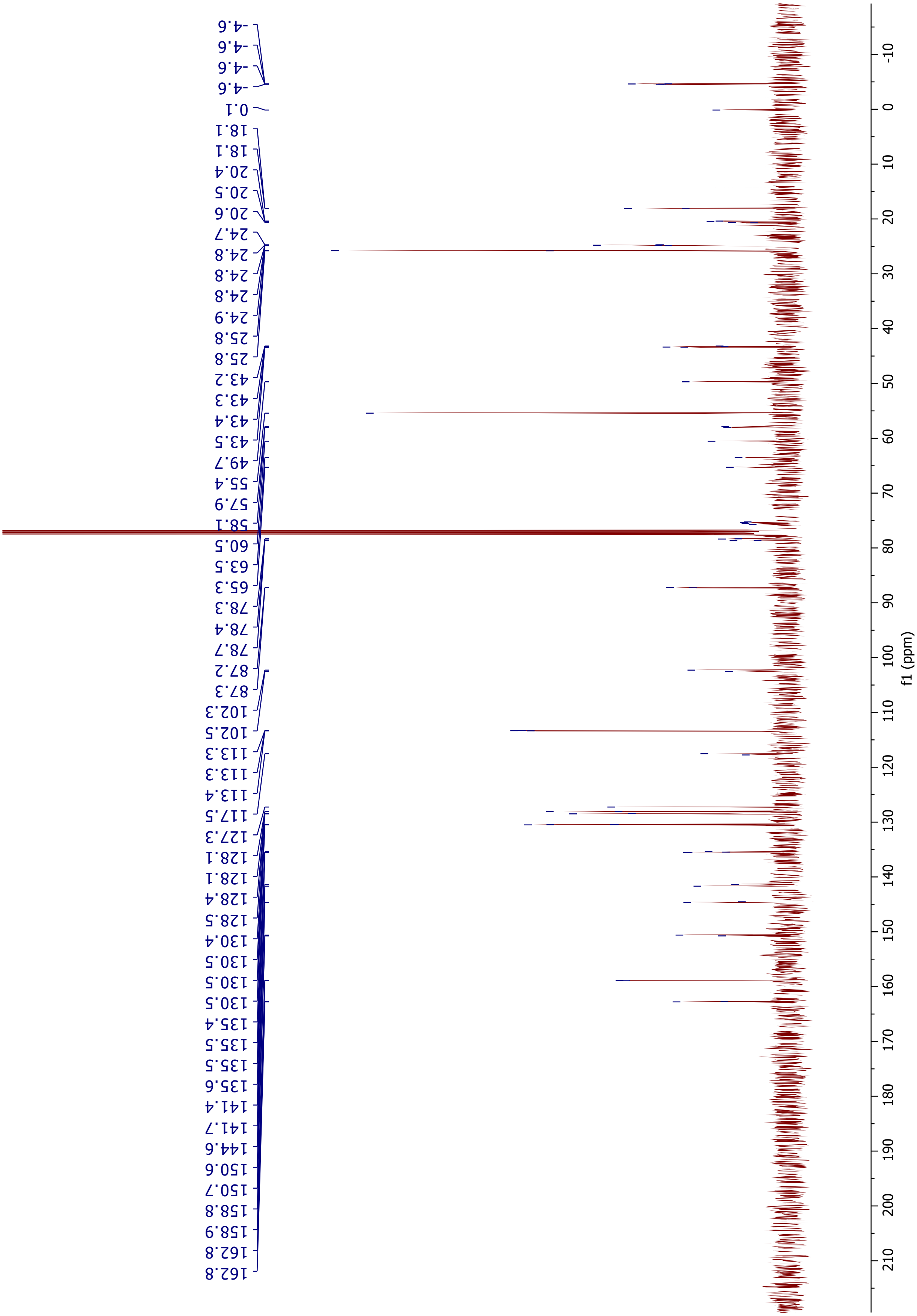

^{31}P NMR (162 MHz, $CDCl_3$)

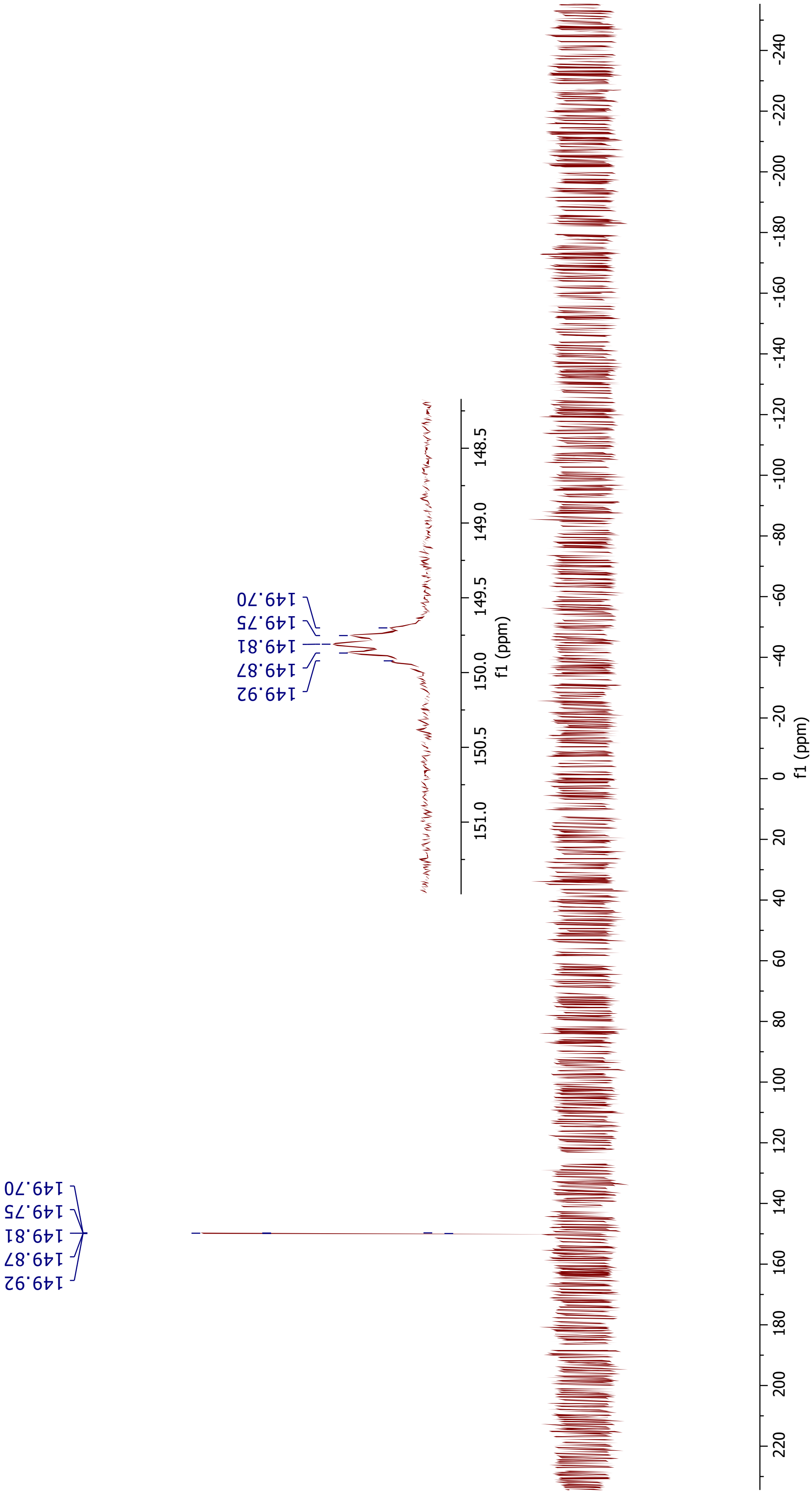

5. References

(1) Benckendorff, C. M. M.; Sanghvi, Y. S.; Miller, G. J. Preparation of a 4'-Thiouridine Building-Block for Solid-Phase Oligonucleotide Synthesis. *Curr. Protoc.* **2023**, *3*, e878.

(2) Mashelkar, K. K.; Byun, W. S.; Ko, H.; Sung, K.; Tripathi, S. K.; An, S.; Yum, Y. A.; Kwon, J. Y.; Kim, M.; Kim, G.; et al. Discovery of a Novel Template, 7-Substituted 7-Deaza-4'-Thioadenosine Derivatives as Multi-Kinase Inhibitors. *Pharmaceuticals* **2021**, *14*, 1290.
